# Feasibility of hearing aid gain self-adjustment using speech recognition

**DOI:** 10.7776/ASK.2022.41.1.076

**Published:** 2022-01-31

**Authors:** Donghyeon Yun, Yi Shen, Zhuohuang Zhang

**Affiliations:** 1 Department of Speech, Language and Hearing Sciences, Indiana University Bloomington; 2 Department of Speech and Hearing Sciences, University of Washington; 3 Department of Computer Science, Indiana University Bloomington

**Keywords:** Hearing aid gain self-adjustment, Method of adjustment, Listening preference, Monte-Carlo simulation, 43.71.An, 43.71.Gv, 보청기 이득 자가 조절, 조절 방법, 선호 청취, Montel-Carlo 시뮬레이션

## Abstract

Personal hearing devices, such as hearing aids, may be fine-tuned by allowing the users to conduct self-adjustment. Two self-adjustment procedures were developed to collect the listener preferred gains in six octave-frequency bands from 0.25 kHz to 8 kHz. These procedures were designed to allow rapid exploration of a multi-dimensional parameter space using a simple, one-dimensional user control interface (i.e., a programmable knob). The two procedures differ in whether the user interface controls the gains in all frequency bands simultaneously (Procedure A) or only the gain in one frequency band (Procedure B) on a given trial. Monte-Carlo simulations suggested that for both procedures the gain preference identified by simulated listeners rapidly converged to the ground-truth preferred gain profile over the first 20 trials. Initial behavioral evaluations of the self-adjustment procedures, in terms of test-retest reliability, were conducted using 20 young, normal-hearing listeners. Each estimate of the preferred gain profile took less than 20 minutes. The deviation between two separate estimates of the preferred gain profile, conducted at least a week apart, was about 10 dB ~ 15 dB.

## 초 록

보청기와 같은 청력 보장구는 자가 이득 조절을 통한 미세 조절이 가능 할 수 있다. 0.25 kHz ~ 8 kHz 옥타브 주파수 밴드의 대상자 선호 이득 측정을 위해 두 종류의 자가 이득 처방 절차가 개발 되었다. 이들 절차는 일차원 사용자 조절 인터페이스(프로그램된 다이얼)를 이용하여 다차원 변수를 빠르게 획득 할 수 있도록 디자인 되었다. 두 종류의 자가 이득 처방 절차는 사용자 인터페이스가 6개 주파수 밴드의 이득을 동시에 조절 하는지(Procedure A) 혹은 각 주파 수 밴드를 개별적으로 조절 하는지(Procedure B) 에 따라 구분 된다. Monte-Carlo 시뮬레이션은 두 종류의 자가 이득 처방 절차에서 첫 20번의 반복된 시도동안 가상의 실제 선호이득값에 빠르게 수렴 할 수 있음을 보여 줬다. 20명의 젊은 정상 청력인을 대상으로 두 종류의 자가이득 처방 절차에 대한 행동 데이터 평가가(실험-재실험에 관한 신뢰도) 이뤄 졌다. 선호 이득 측정은 20 min 미만의 시간이 소요 되었다. 최소 일주일 이상 간격을 두고 측정된 두 번의 사용자 선호 이득의 평균 제곱근 편차는 대략 10 dB ~ 15 dB 수준이었다.

## I. Introduction

Listeners with hearing loss can often benefit from amplification of the acoustic inputs into the ear, which is clinically achieved by hearing aids. Hearing aids separate the input signal into various frequency bands and provide different amounts of gain for different bands depending on the listener’s degree of hearing loss in these bands. A number of prescription procedures are currently used clinically, including NAL-NL2, DSL, CAM2, etc..^[1–3]^ These procedures are mainly based on audibility, but some of them also take loudness comfort into the consideration. One problem of these procedures is that they would prescribe the same amount of gain as long as the hearing thresholds are the same across listeners. This limits the capability of the prescribed amplification to fit individual listener’s needs,^[4]^ and audiologist often do not have a justifiable rationale for adjusting the gain prescription to improve either speech understanding outcome and user’s satisfaction.

Client-based fine-tuning the amplification profiles may be a useful approach to meet individual hearing-aid users’ needs.^[5]^ Hearing aid users are frequently unsatisfied from standard fitting in terms of loudness comfort. Allowing the users to self-adjust the gain, many people can do it reliably, and a subset of them reported improved satisfaction. Mackersie *et al*.^[6]^ also showed that user-adjusted gain profile can achieve comparable speech audibility as provided by standard formula such as NAL-NL2. However, the success of these self-adjustment schemes is highly dependent on whether users can perform the adjustment without errors.^[7]^

There are a number of approaches that have been adopted to obtain estimates of user preferred hearing aid parameters,^[5,7–17]^ and two major approaches are commonly adopted. First, Kuk and Lau^[14]^ introduced a paired-comparison and simplex adaptive procedure, in which subjects are required to compare two speech passages (i.e., a reference and a comparison stimulus) embedded in a multi-talker babble noise on every trial and choose the one they prefer. The speech passages are amplified using two frequency bands. The gains in the two frequency bands are adaptively varied according to a heuristic optimization algorithm, namely the simplex algorithm. At the beginning of the procedure, the low- and high-frequency gains are set according to the NAL-R prescription^[18]^ and served as the reference. In a pair of trials, either the high- or low-frequency gain for the comparison stimulus is incremented from the reference, respectively. For each of the high- and low-frequency bands, if the incremented gain is preferred by the subject, it is implemented as the reference in the next pair of trials; otherwise, a gain decrement is applied to the next reference. Across a number of trials, the gains for the reference adaptively varies. When three reversals of the adaptive track are reached, the procedure is terminated, and the last reference was considered as the final estimate. Kuk and Lau^[14]^ showed that listeners’ preferred gain at 1000 Hz and 2000 Hz was correlated with gains prescribed by NAL-R in most conditions.

Second, real-time adjustment of parameters of amplification profile^[6,8,15,19]^ allows listeners to adjust several parameters using a user interface (e.g., programmable knobs or clickable buttons on a computer screen) while listening to the effects of the parameter adjustments in real time. Some examples for the adjusted parameters are low-frequency gain, the slope at which high-frequency gain increases with frequency, and overall level. It has been showed that the speech audibility increases following the self-adjustment procedure in most listeners, comparable to that provided by standardized formulas (e.g., NAL-NL2) and that self-adjusted gain obtained by repeated sessions was relatively consistent.^[6,15]^ When implemented in commercial hearing aids, self-initiated adjustment could occur voluntarily during hearing-aid use, and the listener-adjusted settings are then collected over time to train the devices about the listeners’ preferences.^[17,19,20]^ This allows in-situ adjustments and adjustment separately for various programs/listening environments.

One advantage of paired-comparison over self-adjustment is that the listener’s decision-making process is relatively simple, because the pair of Reference and Comparison stimuli only differ along one stimulus dimension on a given trial, while the simultaneous adjustments along multiple stimulus dimensions sometimes required for the adjustment procedure could be challenging for an inexperienced user to perform.^[11]^ However, paired-comparisons require repeated presenting stimuli in pairs, which may be potentially more time consuming. This may set a limit to the number of parameters for which the user preference can be estimated. The current study proposes a hybrid approach that combines the advantages of the paired-comparison and self-adjustment approaches. For this hybrid approach, listeners make self-directed adjustments to the gain profile along a single stimulus dimension on each trial, i.e., a relatively simple task. The adjusted stimulus dimension is adaptively determined across multiple trials, similar to the simplex algorithm used in the paired-comparison approach. This hybrid approach simplifies the listeners’ task and control interface while allowing in-situ explorations of a high-dimensional stimulus space, making configurations for multiple stimulus parameters (e.g., gain values in multiple frequency bands).

In the following, two procedures developed using the hybrid approach are described. The feasibility of the procedures is then demonstrated using Monte-Carlo simulations. The simulations aim to demonstrate whether the procedures would lead to accurate estimates of the preferred gain profile in six octave-frequency bands. The rate at which the error in the estimated preferred gain profile reduces with the number of trials, i.e., the rate of convergence, is also inspected. Following the simulation results, initial behavioral evaluations of the procedures are also presented, focusing on quantifying their test-retest reliability. Results, in terms of the deviation between two runs of the self-adjustment procedures, are compared between the two procedures and across various stimulus conditions (e.g., speech presented in quiet or in noise, at various signal-to-noise ratios).

## II. Two self–adjustment procedures for estimating the preferred gain profile

Two self-adjustment procedures (Procedures A and B) were developed in the current study (MATLAB based). These procedures provide estimates of listeners’ preferred gain profiles across six octave-frequency bands using a simple control interface (i.e., a programmable knob), resembling an ear-level user control (e.g., control buttons on a hearing aid, located behind the user’s ear). Each gain profile can be considered as one location in a six-dimensional parameter space, with each dimension corresponding to the gain in each of the six frequency bands. The aim of the proposed procedures is to allow the listener to efficiently explore the parameter space and identify the location in the space associated with greatest preference.

During each run of the procedures, the listener adjusts the gain profile while listening to a continuous speech stimulus on each trial. Before the *n*th trial, the preferred gain profile selected by the listener on the last trial (**m**_*n*-1_) is considered as the reference location in the parameter space. Turning the knob causes deviations from the reference location along a linear function that passed through the reference location. The orientation of the linear function was governed by a vector **w**_*n*_, so that:

(1) $$x=w_{\text{n}}\text{z}+\mu_{\text{n}-1},$$

where z (a scalar) is the variable controlled by the knob; the row vector **w**_*n*_ = {*w*_*n*,1_, *w*_*n*,2_, …, *w*_*n*,6_} indicates the direction of the gain adjustment on the *n*th trial. **w**_*n*_ is normalized so that:

(2) $$\sum_{i=1}^{6}w_{n,i}=0,\;\text{and}$$

(3) $$\frac{1}{6}\sum_{i=1}^{6}w_{n,i}^{2}=1.$$

The normalization is implemented to limit variations of sound pressure level across trials. For Procedure A, the elements in **w**_*n*_ are drawn from a six-dimensional Gaussian distribution before normalization. On the other hand, for Procedure B, one element in **w**_*n*_ is randomly selected to be unity while all other elements were set to zero before normalization.

For both procedures, the listener adjusts the knob until the most preferred speech quality was achieved, which is used as the reference location for the following trial (**m**_*n*_). For Procedure A, the progression from **m**_*n*-1_ to **m**_*n*_ could be toward any direction in the parameter space, while for Procedure B, the progression would be along one of the six axes. Over a number of trials, the sequences of reference locations (**m**_1_, **m**_2_, ···, **m**_*n*_) would represent two fundamental forms of random walk (Gaussian random walk for Procedure A and Lattice random walk for Procedure B) under the influence of a gradient field set by the listener’s preference.

For both Procedures A and B, at the beginning of each 50-trial run, the gain in each of the six bands were randomly drawn from a uniform distribution spanning between −30 dB and 30 dB, independently. This gain profile was used as the initial reference location (**m**_0_). The randomization of the initial gain profile was implemented to ensure that the results reported were not limited to a particular choice of the initial gain.

## III. Evaluation of the self–adjustment procedures using monte–carlo simulations

### 3.1 Simulation procedures

To demonstrate that the iterative algorithms in the two procedures can lead to convergent movements of the reference locations towards the preferred gain profile over trials, a series of Monte-Carlo simulations were conducted in MATLAB. Additionally, the simulations were used to determine whether the final estimated preferred gain profile should be based on the last reference location of the procedure or the average across a number of reference locations at the end of the procedures.

In the simulations, the proposed procedures (Procedures A and B) were conducted on simulated listeners. Each simulated listener was specified by a ground-truth preferred gain profile, modeled as a six-dimensional Gaussian distribution. The mean of the distribution indicated the preferred gain of the simulated listener, **m**_ground-truth_, it consisted of six gain values for the six octave-frequency bands, each of which was randomly drawn from a uniform distribution between −30 dB and 30 dB. The preferred gain profiles were randomly determined for the simulated listeners so that the evaluation of the procedures would not depend on a particular ground-truth profile. The variability of the gain preference in each frequency band was determined by a standard deviation, *σ*_0_, and it was assumed to take the same value across the six bands. On each trial of the adaptive procedure, the simulated listener provided a response by drawing a random sample from the Gaussian distribution that was along the specified stimulus dimension [Eq. (1)]. A larger *σ*_0_ value would lead to more varied responses, while a smaller *σ*_0_ value would lead to more consistent responses.

Simulations were conducted for each of Procedures A and B. For each procedure, since the appropriate *σ*_0_ value that represented a human listener was not known *a priori*, simulations were repeated for *σ*_0_ values of 2 dB, 5 dB, 10 dB, and 20 dB. For each *σ*_0_ value, 200 simulated listeners were constructed, and the preferred gain profile was estimated using one run of the procedure from each simulated listener. Each run of the procedure consisted of 50 trials. At the end of each run, the final estimated preferred gain profile was either the reference location collected during the last trial or calculated based on the average of the reference locations across trials, excluding those from the first 10 trials. The exclusion of the first 10 “burn-in” trials was to avoid the influence of the initial reference location (**m**_0_) to the final estimate.

To evaluate the convergence of the procedures toward the ground-truth, the Root-Mean-Square deviation (RMS error) between the reference location identified by the simulated listener on each trial (**m**_*n*_) and the ground-truth preferred gain profile (**m**_ground-truth_) was calculated. To evaluate the appropriate method to derive the final estimate for the preferred gain profile, results from the two methods based on the last reference location and the average of the last 40 trials were compared in terms of RMS error.

### 3.2 Results

**Fig. 1.**
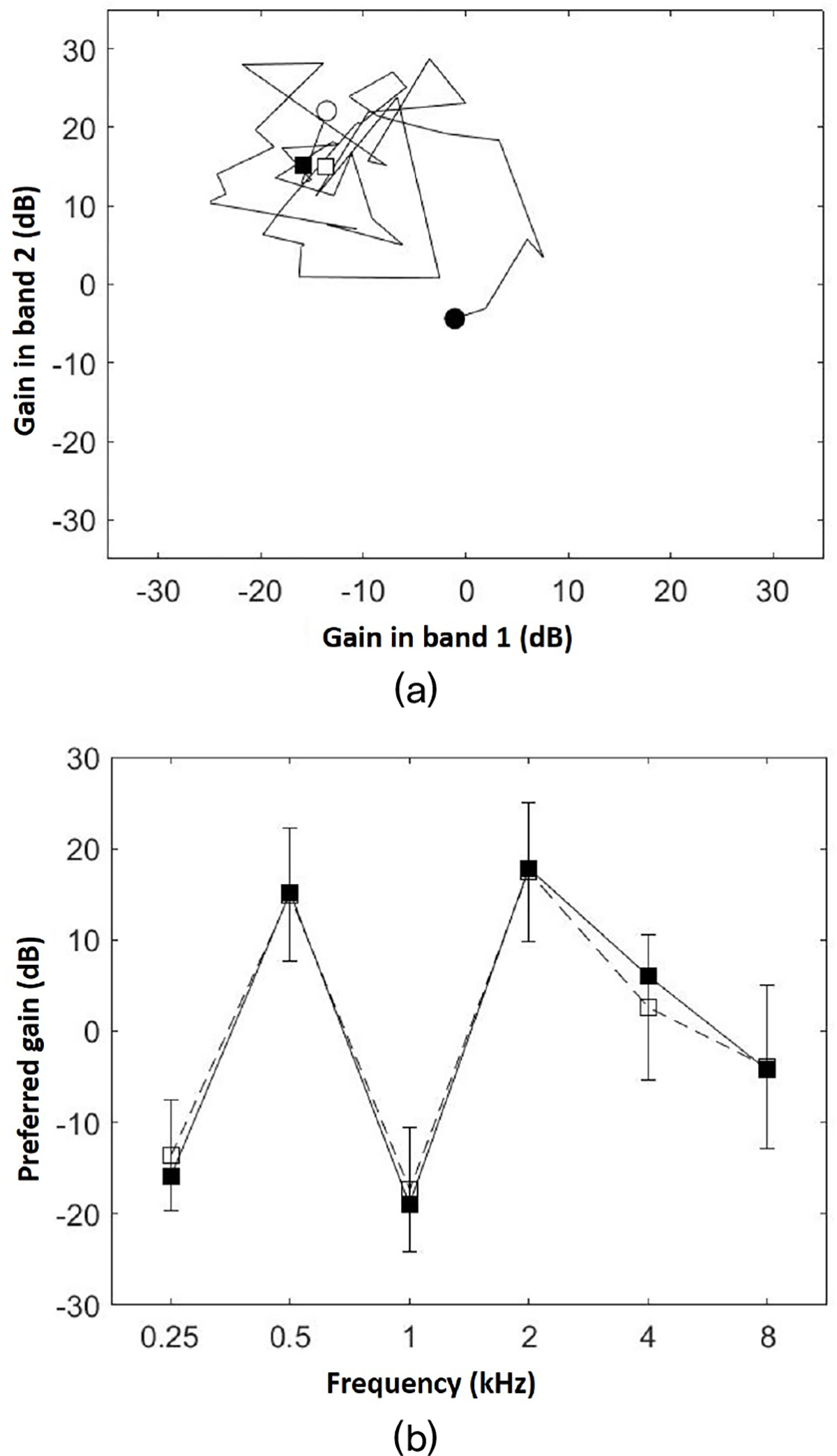
An example simulated run of procedure A. Error bars indicate ± one standard deviation across the last 40 trials.

Fig. 1 illustrates how the reference location converges during one run of Procedure A. The trajectory of the reference location across 50 trials in the six-dimensional parameter space is plotted in Fig. 1(a) (only for the 0.25 kHz and 0.5 kHz bands). In the example shown in the figure, the initial reference location (**m**_0_, filled circle) was randomly determined and was at a certain distance away from the ground truth (**m**_ground-truth_, filled square). As more trials were run, the reference location moved on a trial-by-trial basis, resembling a random walk. The migration of the reference location was towards the ground truth in general and stayed within the neighborhood of the ground truth after the initial couple of trials. Following the procedure, the final estimate of the preferred gain profile was either the last reference location (unfilled circle) or the average of reference locations over the last 40 trials (unfilled square). In this example, the final estimate based on the 40-trial average was closer to the ground truth. Fig. 1-b plots the final estimate based on the 40-trial average (unfilled square) against the ground truth (filled square). The estimated preferred gain profile closely agreed with the ground truth.

**Fig. 2.**
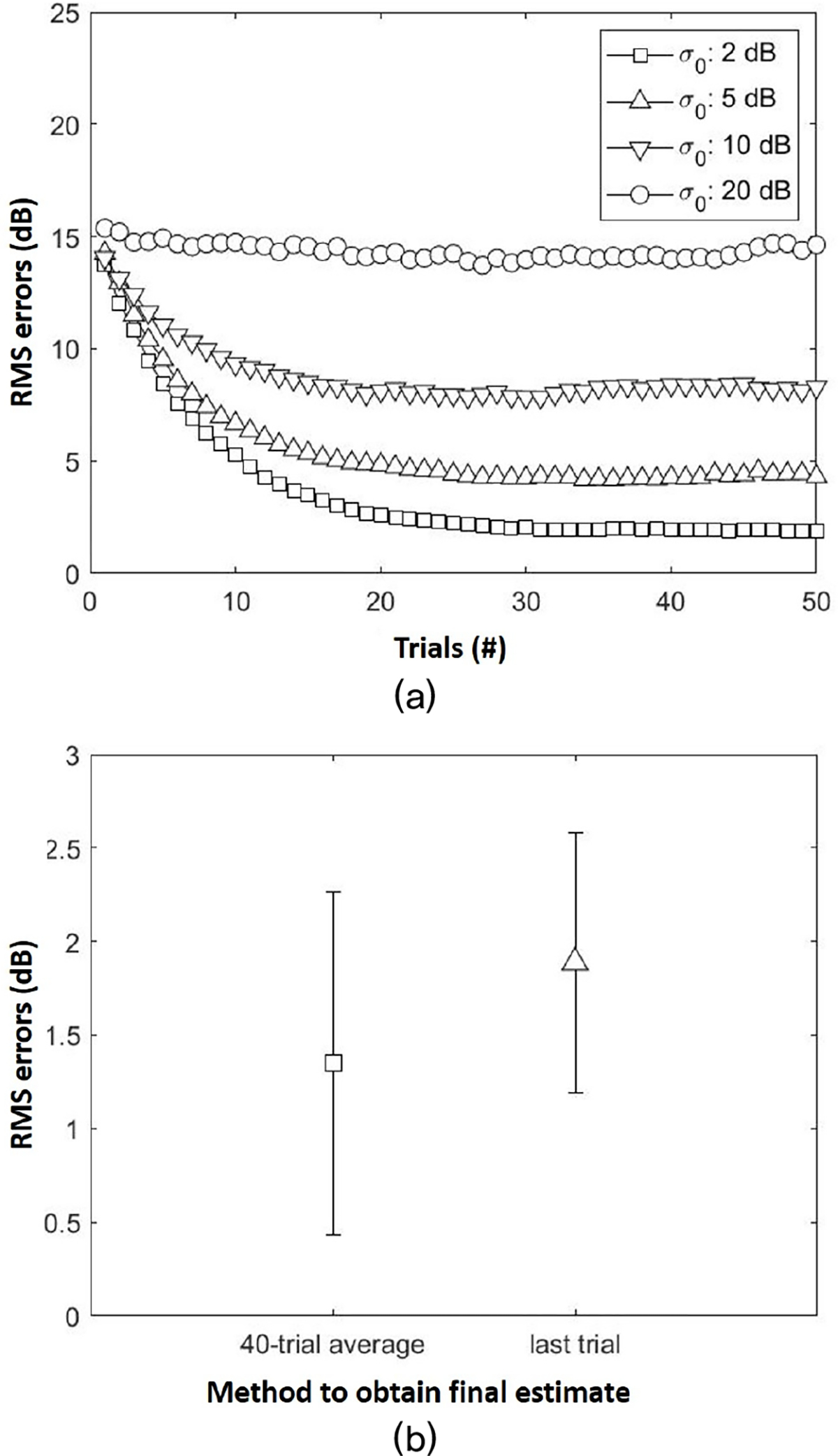
The RMS errors between the preferred gain profile identified by simulated listeners and the ground truth value for procedure A.

Fig. 2-a shows the average RMS errors between the reference location (**m**_*n*_) and ground- truth preferred gain (**m**_ground-truth_) as functions of trial number for Procedure A. The RMS errors was larger for larger values of *σ*_0_. This was expected because the accuracy of the estimated gain preference would be poorer for listeners with greater variability in identifying their preferences. For each *σ*_0_ value, the RMS error decreased as more trials were collected. The RMS error typically (for *σ*_0_ values of 2 dB, 5 dB, and 10 dB) dropped rapidly during the first 20 trials and then reduced at a much more gradual rate for the remainder of the procedure. The same pattern was also observed in Procedure B. Fig. 2(b) compares the RMS errors (*σ*_0_ value of 2) between two methods to obtain the final estimate of the listener’s preferred gain profile. Across the 200 simulated listeners, the RMS error between the final estimate and ground-truth preferred gain profile was smaller when the final estimate was based on the average across the last 40 trials compared to the method based on the last trial. [*t*(199) = 10.46, *p* < .001 for Procedure A, and *t*(199) = 13.59, *p* < .001 for Procedure B]. This result suggests that using the 40-trial average provides a more accurate estimate of the preferred gain profile compared to the last reference location. This estimation method was used for the behavioral evaluations of the self-adjustment procedures.

## IV. Initial behavioral evaluations of the self–adjustment procedures

### 4.1 Methods

Two behavioral experiments were conducted to provide initial evaluation of the test-retest reliability of the two proposed self-adjustment procedures. In Exp. I, two estimates of the preferred gain profiles were conducted at least one week apart and compared, separately for Procedures A and B. In Exp. II, two estimates of the preferred gain profile were conducted using Procedure A in the same test session. Comparing results from the two experiments would help clarify the effect of test interval on test-retest reliability.

A total of 20 listeners participated in this study, 10 of them (0 male and 10 females) participated in Exp. I and the remaining 10 (2 males and 8 females) participated in Exp. II. Their ages ranged between 20 and 31 years (mean age, 23.2 years). All participants had hearing thresholds lower than 10 dB HL across frequencies between 250 Hz and 8000 Hz. The average pure-tone average thresholds (PTAs, across 0.5 kHz, 1 kHz, and 2 kHz) for the left and right ears were 4.7 dB and 5.0 dB HL, respectively. For each listener, the ear with a lower PTA was used as the test ear. All procedures were explained to the listeners, and informed consent was obtained. The experimental protocol was approved by the institutional review board at Indiana University.

Each estimate of the preferred gain profile using either Procedure A or B consisted of 50 trials. A continuous speech stimulus was presented throughout the procedure, and the listener’s task on each trial was to adjust the presented stimulus using a programmable knob until a preferred setting was reached. The speech stimulus was a passage spoken by a male talker in American English, recorded for the measurement of the uncomfortable loudness level during audiometric testing (recorded by Auditec, Inc.). The speech stimulus was presented at 65 dB Sound Pressure Level (SPL), either in quiet or in a simultaneous broadband noise. The background noise was a broadband noise spectrally shaped to match the long-term spectrum of the speech stimulus. The continuously presented speech (in the absence of the background noise) or speech-and-noise mixture (when the background noise was present) was filtered by a bank of one-octave band-pass filters centered at 0.25 kHz, 0.5 kHz, 1 kHz, 2 kHz, 4 kHz, and 8 kHz. Gains were then applied to the filtered signals in the six frequency bands [**x** in Eq. (1)] in real time according to the listener’s adjustment of the knob [*z* in Eq. (1)]. Following the application of the gains, the signals in the individual frequency bands were recombined and presented to the test ear through a soundcard (Microbook II, Mark of the Unicorn, Inc., Cambridge, MA) and a pair of supra-aural headphones (HD280 Pro, Sennheiser electronic GmbH & Co. KG, Hanover, Germany).

Experiment I included two test sessions, at least one week apart. During the first session, the listeners’ preferred gain profiles were estimated using the two procedures. The order in which the two procedures were tested was counter-balanced across listeners. For each procedure, the estimation was repeated for Signal to Noise Ratios (SNRs) of 0 dB, 5 dB, and 10 dB and in the absence of the background noise, conducted in random order. This process was repeated in the second session using independently determined random orders. Experiment II included two runs of Procedure A in the same test sessions.

Additional care was taken to ensure that the listener understood and stayed engaged in the experimental task. Before data collection began, instructions of the experimental task were provided to the listeners in both oral and written format. Demonstrations of how the knob would affect the stimuli were given to the listeners. The listeners were explicitly instructed to explore the knob in both clockwise and counterclockwise directions on each trial. They had up to 15 s to identify the preferred gain profile, otherwise, the gain profile at 15 s following the beginning of the trial would be considered as the estimated gain profile from that trial. The listeners had the opportunity to advance to the next trial before the end of the 15 s interval (by pressing the “OK” button on the knob), however they would need to spend at least 5 s adjusting the gain profile before the advance option was activated. The experimenter monitored the listeners responses closely during the experiment to prevent the listeners advancing through trials without adjusting the knob.

### 4.2 Results

**Fig. 3.**
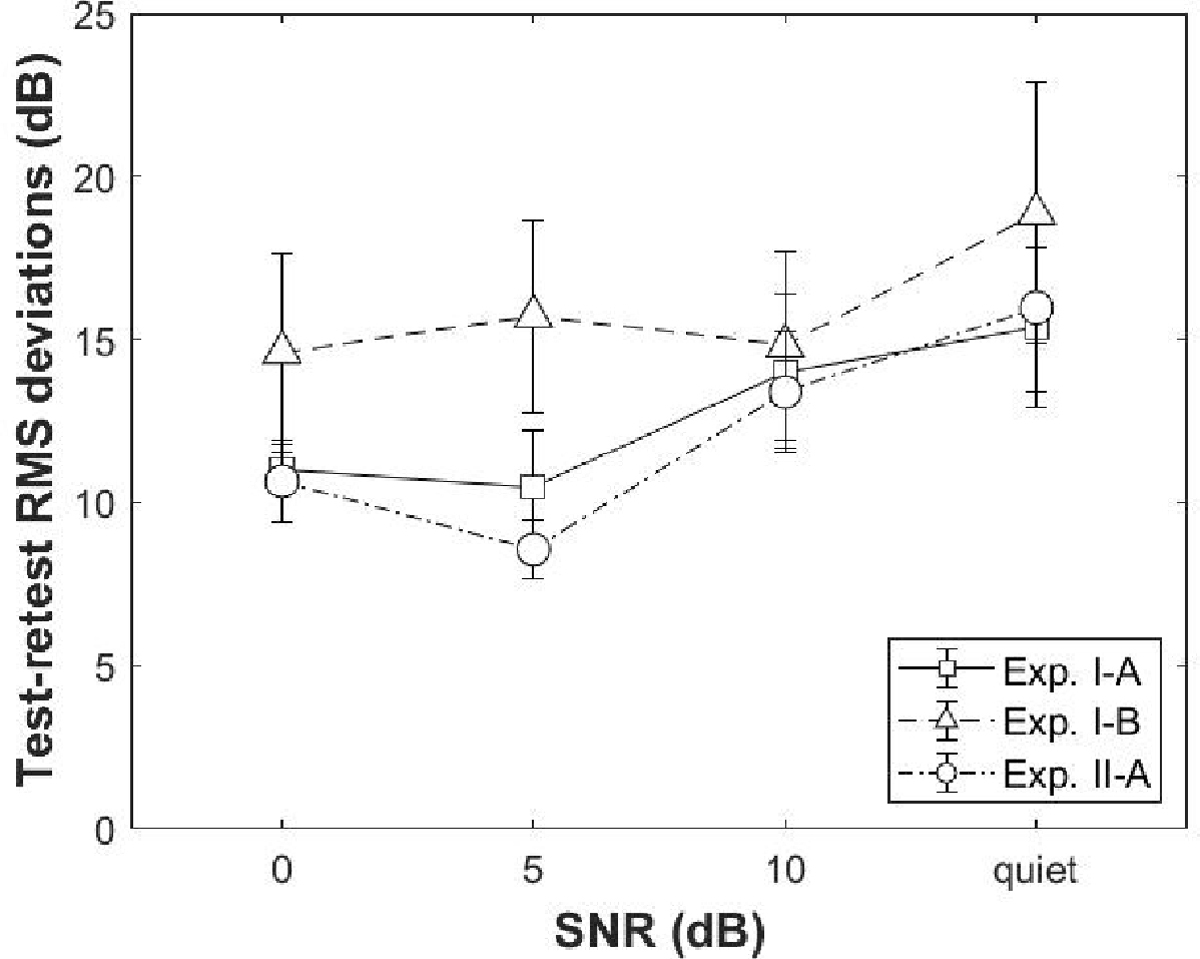
The root–mean–square deviations between the test and retest for the estimated listeners’ preferred gain profiles. Error bars indicate ±one standard error of the mean.

To evaluate the test-retest reliability of the self-adjustment procedures (i.e., Procedures A and B), the deviation between the estimated listener’s preferred gain profiles from the two runs of the same procedure for the same listener and condition were calculated. The root-mean-square value of the deviation across the six octave-frequency bands, i.e., the RMSD, was used as the indicator of the test-retest reliability. Fig. 3 shows the test-retest RMSD for Exps. I (line with squares and line with triangles) and II (line with circles).

For Exp. I, the RMSDs reflected the reliability between two runs of the self-adjustment procedures that were separated by at least a week. The average RMSD across listeners were greater than 10 dB for both Procedures A and B and for all SNRs. A repeated-measures Analysis of Variance (ANOVA) with procedure and SNR as the independent variable and RMSD as the dependent variable found no significant main effect of procedure [*F*(1, 9) = 2.948, *p* = 0.120] or SNR [*F*(3, 27) = 1.417, *p* = 0.259], neither was there a significant interaction between the two factors [*F*(3, 27) = 0.411, *p* = 0.746].

For Exp. II, the RMSDs reflected the test-retest reliability between two runs of Procedure A conducted in close succession. These RMSDs were slightly lower than those from Exp. I, suggesting that poor reliabilities tend to be associated with longer intervals between the test and retest. A repeated-measures ANOVA with SNR as the independent variable and RMSD as the dependent variable found a significant effect of SNRs [*F*(3, 27) = 6.826, *p* = 0.001]. To investigate the effect of SNR further, *post hoc* paired comparisons were conducted. This analysis showed no significant difference in RMSD for all conditions (*p* > 0.05, Bonferroni corrected). There were marginal tendencies that the RMSD was lower for the 5 dB than 10 dB SNR [*t(9)* = −*3.345*, *p* = *0.052*, Bonferroni corrected], and it was lower for the 5 dB SNR than the quiet condition [*t(9)* = −*3.071*, *p* = *0.08*, Bonferroni corrected]. These observed tendencies were in agreement with previous studies, indicating better test-retest consistency in preferred gains under moderate noisy conditions.^[12,15]^

## V. Discussion

The current study presents two adaptive procedures for self-directed adjustment of the gain profile. These procedures aim to enable comprehensive adjustment of the gain preference in six octave-frequency band using a simple, one-dimensional user interface. This is done using an approach in which the listener adjusts the gain profile along one stimulus dimension on each trial and the stimulus dimension is varied from trial to trial.

### 5.1 Accuracy of the self–adjustment procedures

The current Monte-Carlo simulations suggested that the preferred gain profile estimated using this approach typically migrates toward the ground-truth preferred gain profile over trials, resembling a random walk. The accuracy of the estimate is limited by the variability of the listener when identifying the preferred gain. The final estimate of the preferred gain profile may be made more precise by averaging the preferred gain profiles identified by the listener across a number of trials (e.g., the last 40 trials in a 50-trial procedure).

It is worth pointing out that the interpretations of the results from these Monte-Carlo simulations depend on the assumptions used when constructing simulated listeners. In the current simulations, each simulated listener was assumed to have an invariant preferred gain profile, which served as the ground true to evaluate the accuracy of the estimated preferred gain profile. Besides the invariant preferred gain profile, another assumption underlying the simulated listeners was that their identified preferred gains in the six frequency bands have equal variances. This may not be true if human listeners place different emphasis on these frequency bands while making the preference judgement on the speech stimuli as a whole. Additionally, the identified preferred gains in these bands were assumed to be independent from each other. In practice, if a listener prefers higher gains in one frequency band, it is likely that the listener would also prefer higher gains in adjacent bands. For these reasons, there may be an apparent discrepancy between the simulated listeners and human listeners.

### 5.2 Reliability of the self–adjustment procedures

Results from the initial behavioral evaluations (i.e., Exps. I and II) showed that the RMSDs between two runs of the self-adjustment procedures were on the order of 10 dB ~ 20 dB in most test conditions. Similar large intro-subject variability in self-adjusted gain profile has been reported in previous studies. For example, Nelson *et al*.^[15]^ showed that the average differences between test and retest (in the same test session) were 5.6 dB and 6.9 dB for the preferred gains in a low- (0.125 kHz to 1 kHz) and a high-frequency (2 kHz to 8 kHz) band, respectively. To enable comparisons of the current results to those of Nelson *et al*.,^[15]^ the estimated preferred gain profiles from Exp. II (obtained in the same test session) were converted so that each profile contained only two gain values, to resemble those estimated for the low- and high-frequency bands in the study of Nelson *et al*..^[15]^ The gain value in the low-frequency band was the average of the estimated preferred gains from the 0.25 kHz, 0.5 kHz, and 1 kHz bands, while the gain value in the high-frequency band was the average of the estimated preferred gains from the 2 kHz, 4 kHz, and 8 kHz bands. The average test-retest deviations across SNR conditions for the converted preferred gains in the low- and high-frequency bands were 5.43 dB and 5.93 dB, respectively, which were similar to those reported by Nelson *et al*..^[15]^

As another example, Vaisberg *et al*.^[21]^ reported the test-retest reliability for the estimated preferred gains in three frequency bands (0.1 kHz ~ 0.8 kHz, 1 kHz ~ 2.5 kHz, and 3 kHz ~ 10 kHz). These authors showed that between two runs of the self-adjustment procedures conducted in the same test session the preferred gains were within two adjustment steps (less than 12 dB) for 93 %, 83 %, and 71 % of the participants, in the low-, mid-, and high-frequency bands, respectively. To enable comparisons of the current results to those of Vaisberg *et al*.,^[21]^ the estimated preferred gain profiles from Exp. II, were converted so that each profile contained three gain values, to resemble those estimated for the low-, mid- and high-frequency bands in the study of Vaisberg *et al*..^[21]^ The gain value in the low-frequency band was the average of the estimated preferred gains from the 0.25 kHz and 0.5 kHz bands, the gain value in the mid-frequency band was the average of the estimated preferred gains from the 1 kHz and 2 kHz bands, and the gain value in the mid-frequency band was the average of the estimated preferred gains from the 4 kHz and 8 kHz bands. The average test-retest deviations across SNR conditions were within 12 dB for 100 %, 90 %, and 90 % of the participants in Exp. II in the low-, mid-, and high-frequency bands, respectively. These were similar to those reported by Vaisberg *et al*..^[21]^

Therefore, although the procedures used to estimate the user’s preferred gain profile varied across studies, the test-retest reliability reported in these studies were relatively consistent. When the test and retest are conducted in the same test session, the test-retest deviation in the self-adjusted gain is approximately 10 dB for each octave-frequency region. If the proposed self-adjustment procedures were to be applied to hearing-aid fitting, their test-retest reliability, as demonstrated in the current behavioral experiments, is likely to be poorer than the current best clinical practice. In typical clinical practice, the gain profile is prescribed based on the audiogram, the test-retest variability for the real-ear insertion gain typically ranges between 2 dB and 7 dB.^[22]^ A number of reasons may underlie the lack of robust test-retest reliability of self-adjustment procedures. For example, first, it is possible that the preferred gain profile may vary over time, either across trials during the self-adjustment procedures or across multiple runs of the procedures. Second, poor understanding of the task and fatigue may cause performance lapses. Third, the traditional gain prescription is based on audiometric threshold, which is based on a simple detection task. On the other hand, comparing the quality of speech stimuli is a more complex task, involving multiple perceptual dimensions.^[23–25]^ Future research is needed to more precisely define roles of those factors.

## VI. Summary

The current study evaluated the feasibility and the reliability of two self-adjustment procedures to estimate listeners’ preferred gain profiles. Monte-Carlo simulation data showed the feasibility and the time-efficiency of the self-adjustment procedures. Behavioral data (Exps. I and II) showed that the average root-mean-square deviation between test and retest across listeners were approximately 10 dB ~ 15 dB.
